# Parents’ Perceptions and Adherence to Children’s Diet and Activity Recommendations: the 2008 Feeding Infants and Toddlers Study

**DOI:** 10.5888/pcd12.150110

**Published:** 2015-09-24

**Authors:** Ronette R. Briefel, Denise M. Deming, Kathleen C. Reidy

**Affiliations:** Author Affiliations: Denise M. Deming, Kathleen C. Reidy, Nestlé Infant Nutrition Global R&D, Florham Park, New Jersey.

## Abstract

**Introduction:**

Solving the childhood obesity problem will require strategies for changes in policy, the environment, the community, and the family. Filling the data gap for children younger than 4 years could facilitate interventions aimed at this critical age group. The objective of this study was to describe parents’ and caregivers’ perceptions of the healthfulness of their young child’s diet and body weight and to assess their adherence to the American Academy of Pediatrics’ 5-2-1-0 recommendations.

**Methods:**

We conducted a descriptive analysis of parents’ and caregivers’ survey data for 887 infants younger than 12 months, 925 toddlers aged 12 to 23.9 months, and 1,461 preschoolers aged 24 to 47.9 months. Data were from the national, cross-sectional 2008 Feeding Infants and Toddlers Study (FITS).

**Results:**

Most parents considered their child’s weight to be about right but were more likely to think their child was underweight (8%–9%) than overweight (2%–3%). Most parents thought their child consumed enough fruits and vegetables: however, only 30% of preschoolers met the recommendation for 5 daily servings. Only 2% of toddlers met the recommendation for no screen time, whereas 79% of preschoolers met the recommendation to limit daily screen time to 2 hours or less. About 56% of toddlers and 71% of preschoolers met the recommendation of at least 1 hour of daily outdoor play. About 56% of toddlers and 52% of preschoolers met the recommendation to limit consumption of sugar-sweetened beverages.

**Conclusion:**

The FITS 2008 findings underscore the ongoing need for research on policies and strategies to prevent childhood obesity from infancy through preschool. Health care providers can play a vital role because they are an important and early point of contact for parents.

## Introduction

Childhood obesity threatens the growth, development, and well-being of children (1,2). National data indicate that, from 2011 through 2012, 8.1% of infants and toddlers (birth through 23.9 months) had high body weight for recumbent length, and 8.4% of preschoolers (aged 2–5 years) were obese (body mass index ≥ the 95th percentile for age and sex based on the 2000 Centers for Disease Control and Prevention growth charts) (3). Several parental practices in early childhood are important contributors to prevention or treatment of childhood obesity: the timing of the introduction of solid foods, the types and amounts of foods consumed, and time spent on play and sedentary activities (1,2,4–8). Building on the evidence for childhood obesity prevention, the American Academy of Pediatrics (AAP) initiated its 5-2-1-0 campaign in 2009, which consisted of the following recommendations for children:

- 5: Eat 5 servings of fruits and vegetables a day.
- 2: Limit screen time (using computers, playing video games, and watching television, videos, or DVDs) to 2 hours a day. Children younger than 2 should have no screen time.
- 1: Strive for at least 1 hour of physical activity a day.
- 0: Limit consumption of sugar-sweetened drinks (9,10).

This study’s objective was to describe parents’ perceptions of their young child’s diet and weight status and to assess their adherence to AAP’s 5-2-1-0 recommendations by using a national US data set, the 2008 Feeding Infants and Toddlers Study (FITS) (9–12). Understanding parents’ practices with regard to their children’s diet and physical activity — and their perceptions about those practices — is the first step in identifying issues to be addressed in order to reduce children’s risk of obesity later in life. The FITS 2008 findings help fill a national data gap on diet and physical activity behaviors of children younger than 4 years and inform the Dietary Guidance Development Project for Infants and Toddlers from Birth to 24 Months and Pregnancy (13), a federal initiative to synthesize scientific data and develop dietary guidelines for children under age 2.

## Methods

The FITS 2008 was a cross-sectional study of a national random sample of US children from birth through 4 years of age living in one of the 50 states or the District of Columbia. The study was designed to obtain information on the diets and related behaviors of US infants, toddlers, and preschoolers from birth through 47 months with a required prestudy dietary intake sample size of 3,200 (12). The sample frame, recruitment, and data collection procedures are described elsewhere (12). In brief, 2 commercial sample frames were used to identify US households with a child under age 4 years. Data were weighted to reflect vital statistics birth data for 2008 (12). All survey instruments and procedures were approved by Mathematica’s independent institutional review board at the time of the study (Public/Private Ventures in Philadelphia, Pennsylvania) and are available at (http://medical.gerber.com/NutritionEducation/FITSStudy.aspx). All participants received written information on the study, understood that participation was voluntary, and were assured of the confidentiality of the data.

The FITS 2008 consisted of up to 3 telephone interviews conducted from June 2008 through January 2009: a recruitment interview to collect household and child characteristics (including the child’s physical activity and screen time) and an interview about diet consisting of a 24-hour diet recall and questions about breastfeeding, the introduction of foods, and parents’ perceptions of their child’s diet and weight status. A representative 25% subsample had a second 24-hour diet recall, which was used to estimate the population’s usual intake of fruit and vegetables.

Before the diet interview, the main caregiver, usually the mother, was mailed a packet of materials consisting of a letter describing the study, a food model booklet, a ruler, a liquid measuring cup with instructions for use, and instructions for reporting foods the child consumed at childcare outside the home. Parents who responded received $20 each as an incentive for participating in the first diet interview and $10 for participating in the second. Diet interviews were conducted in English or Spanish by interviewers trained and certified to collect 24-hour diet recalls, by using the study protocol and the University of Minnesota’s Nutrition Data System for Research 2008 (http://www.ncc.umn.edu/). Diet recalls were collected for all days of the week to reflect weekday and weekend food intake at the group level.

Sugar-sweetened beverages were defined as carbonated soft drinks, fruit-flavored drinks, sports drinks, and other beverages with added sugars. Quantities of fruits and vegetables, including juices, were translated to cup equivalents by using the US Department of Agriculture’s (USDA’s) MyPyramid Equivalents Database, version 2.0 (14). The *SAS Macros for the NCI Method* (http://riskfactor.cancer.gov/diet/usualintakes/macros.html) and guidelines developed by the National Cancer Institute were used to estimate distributions of usual intake of total fruits and vegetables from all sources, including 100% juices. Foods were broken into their component parts, and any fruit or vegetable component of a food was assigned to the fruit or vegetable group. For instance, the tomato sauce in pizza counted as a vegetable as did the potato in French fries.

The standard used to assess meeting the recommendation of 5 servings a day was the equivalent of 2.5 cups of total fruits and vegetables combined for the 1,000-calorie USDA Food Pattern. This 1,000-calorie level is recommended for all 2-year-olds and most 3-year-olds on the basis of a sedentary level of physical activity (14). Because the dietary guidelines and associated food patterns are not yet developed for children under age 2 years, it is not possible to estimate adherence to the AAP fruit and vegetable recommendations for this group; therefore, our analysis is restricted to 2-year-olds and 3-year-olds. Daily physical activity (ie, sports activities, outdoor play) and daily screen time were calculated on the basis of the reported frequency and time spent (in hours or minutes during the previous week).

### Statistical analyses

Sample sizes for this analysis were 382 infants aged 0 to 5 months, 505 infants aged 6 to 11 months, 925 toddlers aged 12 to 23 months, 736 two-year-olds aged 24 to 35 months, and 725 three-year-olds aged 36 to 47 months. Excluded from the dietary analysis were 2- and 3-year-olds with missing data on household income (n = 138), an important covariate in the models to estimate usual intake when using the SAS macros developed by the National Cancer Institute (http://riskfactor.cancer.gov/diet/usualintakes/macros.html).

All analyses used sample weights that reflected the age and racial/ethnic distribution of US children from birth to 4 years in 2008 and accounted for nonresponse and coverage of the target population. Estimates (means, proportions) were calculated by using SAS version 9.1.3 (SAS Institute) and the appropriate sample weights and design effects. Standard errors were calculated by using SUDAAN release 9 (RTI Institute).

## Results

### Sample characteristics

Among sampled households that could be located by telephone to verify an age-eligible child (7,232), the completion rate for the recruitment interview was 60% (n = 4,339). Response rates are comparable with other large-scale national surveys conducted by telephone (15). About half (53%) of the children were male; 56% were non-Hispanic white, 21% were Hispanic, 14% were non-Hispanic black, and 8% were other race/ethnicities or multiple races. Compared with the 2008 Current Population Survey, the FITS 2008 household income distributions were similar, but the FITS 2008 respondents were more likely to be college-educated (US Census, 2010) (12). About 30% of the sample participated in the Special Supplemental Nutrition Program for Women, Infants, and Children, and 35% were first-born children. Half of the mothers were employed (51%), almost half had college degrees (46%), and nearly half (48%) of the children were in childcare outside the home. Other sample characteristics are described in previous publications (12). (Unweighted characteristics are available from R.R.B. on request.)

### Parents’ perceptions of child’s weight status and diet

Parents were asked to report whether their child was overweight, about the right weight, or underweight. Most parents reported that their child was “about the right weight” (Table 1). Parents of the older infants, toddlers, and young preschoolers were more likely to report their child was underweight than overweight.

**Table 1:** Parents’ (n= 3,378) Perceptions About Their Child’s Weight and Diet and Reported Dietary and Physical Activity Practices, 2008 Feeding Infants and Toddlers Study

Survey Questions and Answers (Number Surveyed)	Infants 0–5.9 Months^a^	Infants 6–11.9 Months^a^	Toddlers (12–23.9 Months)^a^	2-Year-Olds (24–35.9 Months)^a^	3-Year-Olds (36–47.9 Months)^a^	All Preschoolers (24–47.9 Months)^a^
	% (SE)	% (SE)	% (SE)	% (SE)	% (SE)	% (SE)
**Do you consider your [child] now to be — (n = 3,378)**						
Overweight?	2.0 (0.75)	1.7 (0.56)	2.3 (0.65)	2.0 (0.87)	2.5 (0.67)	2.2 (0.55)
Underweight?	1.5 (0.65)	4.4 (1.24)	8.1 (1.32)	8.9 (1.71)	8.3 (1.42)	8.6 (1.11)
About the right weight?	96.5 (0.98)	93.9 (1.36)	89.6 (1.45)	89.1 (1.90)	89.2 (1.56)	89.1 (1.23)
**Do you consider [child’s] diet to be: (n = 3,377)**						
Very healthy?	96.9 (1.08)	80.1 (3.26)	57.7 (2.54)	42.6 (3.19)	33.9 (2.98)	38.3 (2.19)
Somewhat healthy?	3.1 (1.08)	19.8 (3.26)	41.7 (2.53)	55.5 (3.18)	63.0 (3.00)	59.2 (2.19)
Not too/not at all healthy?	0 (0.0)	0.1 (0.15)	0.6 (0.43)	1.9 (0.74)	3.1 (0.95)	2.5 (0.60)
**Do you think your child gets enough fruit and vegetables in diet? (n=3,372)**						
Yes	35.2 (3.96)	82.8 (2.62)	84.0 (1.87)	76.8 (2.58)	72.0 (2.75)	74.4 (1.89)
No	4.3 (1.22)	7.8 (2.20)	16.0 (1.87)	22.7 (2.55)	27.5 (2.75)	25.1 (1.88)
Doesn’t eat fruits or vegetables	60.5 (4.01)	9.4 (1.54)	0 (0.00)	0.5 (0.51)	0.5 (0.29)	0.5 (0.29)
**How many nights a week does your family typically sit down together to have dinner as a family? (n=3,376)**						
Every night	49.8 (4.10)	45.4 (3.75)	50.0 (2.67)	49.3 (3.21)	49.4 (3.02)	49.4 (2.20)
5 or 6 nights	24.1 (3.09)	23.3 (2.51)	22.7 (2.15)	23.5 (2.34)	24.4 (2.28)	23.9 (1.60)
3 or 4 nights	14.9 (2.78)	18.1 (2.93)	18.3 (2.01)	16.2 (2.61)	17.2 (2.29)	16.7 (1.74)
1 or 2 nights	7.0 (1.52)	9.1 (2.10)	5.7 (1.15)	7.3 (1.79)	7.0 (1.99)	7.2 (1.34)
Never	4.1 (2.22)	4.1 (1.05)	3.2 (0.80)	3.7 (1.67)	2.0 (0.95)	2.9 (0.96)
**About how often does [child] eat food from a fast food restaurant? (n=3,376)**						
Never eats fast food	99.9 (0.07)	83.5 (2.74)	25.5 (2.21)	6.8 (1.23)	5.1 (1.10)	6.0 (0.83)
Less than once per month	0.0 (0.00)	0.8 (0.49)	6.9 (1.20)	6.1 (1.74)	3.9 (1.31)	5.1 (1.10)
1-3 times a month	0.1 (0.07)	8.8 (2.24)	31.3 (2.50)	36.2 (3.09)	31.8 (2.64)	34.0 (2.04)
Once a week	0.0 (0.00)	5.2 (1.92)	24.5 (2.37)	31.1 (2.79)	34.0 (3.04)	32.5 (2.07)
Twice a week	0.0 (0.00)	1.7 (0.77)	8.6 (1.34)	9.7 (1.51)	17.8 (2.39)	13.7 (1.43)
Three or more times per week^b^	0.0 (0.00)	0.0 (0.00)	3.2 (0.94)	10.1 (2.60)	7.3 (1.63)	8.7 (1.55)
**Is your child involved in gymnastics, dance, swimming, or some other type of athletic activity or organized sport? (n = 3,250)**	—c	—c	15.3 (2.24)	24.4 (2.65)	38.3 (3.01)	32.1 (2.07)
**If *yes* to question above, how many times per week does child participate in these activities? (n = 562)^d^**						
Less than once per week	—c	—c	1.1 (0.67)	0	0	0
Once a week	—c	—c	35.8 (8.27)	48.7 (6.56)	50.8 (4.85)	50.0 (3.90)
Twice a week	—c	—c	31.2 (8.43)	23.7 (6.19)	20.6 (3.82)	21.6 (3.30)
Three times a week	—c	—c	13.5 (5.67)	11.1 (2.99)	15.5 (4.68)	14.0 (3.28)
Four or more times a week	—c	—c	18.4 (4.53)	16.5 (4.01)	13.2 (2.80)	14.3 (2.30)
Child plays outside (n = 3,250)	—c	—c	95.0 (1.19)	98.4 (0.84)	98.9 (0.46)	98.6 (0.45)
**Plays outside for 1 h or more per day (n = 3,250)**	—c	—c	56.2 (2.69)	68.1 (3.01)	72.6 (2.99)	70.6 (2.12)
**Child plays video or computer games (n = 3,250)**	—c	—c	4.5 (1.07)	12.0 (2.23)	36.3 (3.08)	25.5 (2.04)
**Child watches TV, videos, or DVDs (n = 3,250)**	—c	—c	74.3 (2.37)	94.8 (1.55)	97.6 (0.69)	96.4 (0.79)
Child watches TV or videos in the room where he/she sleeps (n = 2,067)^e^	—c	—c	14.9 (2.24)	16.7 (2.39)	24.8 (2.87)	21.3 (1.94)

Abbreviation: —, does not apply; SE, standard error.

a Based on age at recruitment. Totals may not sum to 100% due to rounding.

b Maximum response was 7 times per week.

c Does not apply. These questions were asked only of parents and caregivers of children aged 12–47.9 months and not parents of infants.

d Excludes children whose parents reported that they did not engage in any activities listed in the activities question.

e Excludes children whose parents said their children did not watch television, videos, or DVDs; percentages are of children who watch in their bedroom among those who watch any television, videos, or DVDs.

As reported by parents in the FITS 2008, the proportion of children with a very healthy diet significantly declined from infancy to the preschool period. Almost all (97%) parents of the younger infants reported their child’s diet was “very healthy” compared with 34% of parents of 3-year-olds (Table 1). Most parents thought that their child’s diet contained enough fruits and vegetables (83% of parents of the older infants, 84% of parents of toddlers, and 74% of parents of preschoolers).

### Parents’ reports of child’s diet and physical activity 

#### Diet

In addition to its 5–2-1–0 program, the AAP encourages families to eat meals regularly as a family and to limit consumption of fast food (9,10). Most parents of toddlers and preschoolers reported that they ate dinner together on most nights during the week (about half every night and one-fourth on 5 or 6 nights) (Table 1). About 10% reported that they ate dinner as a family 1 or 2 nights (7%) or never (3%). The frequency of any fast food consumption increased with age, from about 17% for children aged 6 to 11 months to 95% for 3-year-olds. Parents reported that more than one-third (37%) of toddlers and more than half (55%) of preschoolers ate fast food 1 or more times per week; 9% of preschoolers did so 3 or more times per week.

### Physical activity and screen time

About 15% of toddlers and 32% of preschoolers were involved in some sort of organized activity or sport (Table 1). Among those who were involved in an organized activity, the frequency of participation ranged from once a week to 4 or more times a week. In addition, FITS 2008 parents reported that more than 95% of their toddlers and 99% of preschoolers played outside, and about 56% of toddlers and 71% of preschoolers had at least 1 hour of daily play outside.

The AAP recommends that children limit daily media exposure and screen time (9,10,16,17). The FITS 2008 data show that watching television, videos, and DVDs is a prevalent behavior by age 1 year; 74% of toddlers watched TV, videos, or DVDs (Table 1). Only 2% of toddlers in the FITS 2008 met the AAP recommendation for no screen time; about 60% had less than 1 hour per day (Figure). About 17% of 2-year-olds and 24% of 3-year-olds exceeded the AAP recommendation for no more than 2 hours of (quality) combined screen time. For preschoolers aged 24 to 47.9 months, 21% (SE, 1.84) exceeded 2 hours of average daily screen time (data not shown in figure). About 2.5% (SE 0.78) of toddlers, 2.4% (SE, 0.68) of 2-year-olds, and 4.6% (SE, 1.21) of 3-year-olds watched more than 4 hours of television, videos, or DVDs daily (data not shown in table or figure). Among children who watched any television, videos, or DVDs, 15% of toddlers and 21% of preschoolers did so where they slept (Table 1).

**Figure:**
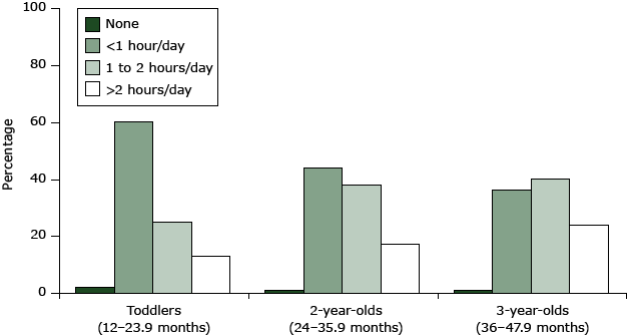
Total daily screen time from computer, video games, television, videos, and DVDs increases with age among toddlers and preschoolers (n = 2,093) according to data from the 2008 Feeding Infants and Toddlers Study (9–12). Total screen time is categorized as none, less than 1 hour a day, 1 to 2 hours a day, and more than 2 hours a day. Children whose records were missing data on screen time were excluded from the analysis. Totals may not sum to 100% because of rounding. **Table:** 

Daily screen time	Toddlers	Two-year-olds	Three-year-olds^a^
**None**	**2**	**1**	**1**
**<1 hour/day **	**60**	**44**	**36**
**1-2 hours/day**	**25**	**38**	**40**
**More than 2 hours/day **	**13**	**17**	**24**

^a^ Does not total 100 because of rounding.

#### Consumption of fruits, vegetables, and sugar-sweetened beverages

Of the 4,339 eligible respondents who completed the recruitment interview and whose children were still age-eligible to participate in a 24-hour diet recall interview, 77 opted out of the diet recall interview, and 884 did not complete the interview, leaving a total of 3,378 (78% response rate). Of the 3,378, 104 had children who had passed the study’s age limit by the time of the interview (ie, the child was 4 years or older). One recall interview was excluded from dietary analysis as an outlier for a total of 3,273 children with eligible diet recalls (12). Table 2 shows the intake distribution of total fruits and vegetables by preschoolers and the percentage who met the recommended 5 servings or more per day. Reported consumption levels were far below these recommended levels; only about 30% of preschoolers met the 5-a-day recommendation.

**Table 2:** Usual Distribution of Preschoolers’ (n = 1,323) Intake of Total Fruit and Vegetables, 2008 Feeding Infants and Toddlers Study

Age, months	n	Mean (SE)	Percentile Value (Cup Equivalents)					Percentage Meeting Recommended 5 or More Daily Servings (SE)^a^
			10th	25th	50th	75th	90th	
24–35.9	666	2.17 (0.03)	1.23	1.62	2.10	2.64	3.19	30.6 (3.46)
36–47.9	657	2.06 (0.036)	1.00	1.40	1.94	2.58	3.27	27.6 (2.64)
Total 24–47.9	1,323	2.12 (0.023)	1.13	1.51	2.03	2.63	3.25	29.6 (2.20)

a Standard error calculation was based on 1-day mean to account for design effects. Nutrition standards are not yet established for children under age 2.

Children in all age groups consumed some amount of sugar-sweetened beverages (Table 3), but consumption was greatest among the oldest children with 48% of 3-year-olds consuming sweetened beverages. Fruit-flavored beverages were the largest share of total sugar-sweetened beverages. Nine percent of 3-year-olds (roughly 1 in 10) consumed carbonated soda on any given day (ie, on a randomly selected day in the United States). About 92% of the older infants met the AAP recommendation of avoiding sugar-sweetened beverages; but only about half (54%) of preschoolers met this recommendation.

**Table 3:** Percentage of Children (n = 2,891) Consuming Sugar-Sweetened Beverages at Least Once a Day, 2008 Feeding Infants and Toddlers Study

Sugar-Sweetened Beverage Consumption	Infants (6-11.9 Months), n = 505	Toddlers (12–23.9 Months), n = 925	2-Year-Olds (24–35.9 Months), n = 736	3-Year-Olds (36–47.9 Months), n = 725	All Preschoolers (24–47.9 Months), n = 1,461
	% (SE)	% (SE)	% (SE)	% (SE)	% (SE)
Any type of sugar-sweetened beverage^a^	7.9 (2.6)	27.8 (2.5)	43.9 (3.2)	48.3 (3.0)	46.1 (2.2)
Fruit-flavored drinks	5.7 (2.5)	20.2 (2.2)	33.2 (3.1)	36.1 (3.0)	34.6 (2.2)
Carbonated sodas	1.1^b^ (0.8)	4.6 (1.4)	7.2 (1.7)	9.2 (1.4)	8.2 (1.1)
Sweetened teas and coffees	0 (0.0)	2.7 (0.8)	7.0 (2.0)	7.6 (1.9)	7.3 (1.4)
Sports drinks	0.9^b^ (0.6)	1.6^b^ (0.5)	2.6^b^ (1.3)	1.0^b^ (0.5)	1.8^b^ (0.7)

Abbreviation: SE, standard error.

a Based on a single 24-hour dietary recall and age at dietary interview. Data are not presented for infants from birth to 5.9 months because of low prevalence, but the point estimate for any type of sugar-sweetened beverage consumption is 0.6% among 4- to 5.9-month-olds. All reports in this age group were fruit-flavored drinks.

b Point estimate is imprecise because of small sample size.

### Discussion

National data from the FITS 2008 show that parents of toddlers and preschoolers correctly perceive that their child’s diet is less healthful than parents of infants perceive their child’s diet to be. But some parents do not recognize when their child is overweight, and some parents even inappropriately perceive that their child is underweight. Parents of young children in the FITS 2008 were more likely to report their children to be underweight than overweight. Although we could not compare this assessment with the child’s actual measured weight, this finding is inconsistent with national data for 2007–2008 of measured heights and weights in this age group (10.1% of children aged 2–5 y were obese) (18). About 2% to 3% of parents perceived their child as overweight (ie, the survey question asked about overweight not obese), but this may be an underestimate of overweight; previous US studies found that parents were often unaware their child was overweight or obese (19,20). With the increased prevalence of obesity among both children and adults in the United States, societal norms may have shifted so that some parents no longer recognize that their child’s weight is not at a healthy level (1,19). Additionally, there are cultural factors such as the belief that a “chubby baby is a healthy baby” that are still prevalent and may interfere with parents’ recognizing their young child as overweight (21).

Parents’ perceptions of their child’s diet and the importance of diet quality in determining health are important factors in whether parents strive to meet dietary recommendations for their children (20). The FITS 2008 data presented here show that parents’ perception of the quality of their children’s diets is inconsistent with respect to recommended levels of fruit and vegetable consumption. Only a third of preschoolers met the recommended 5 or more daily servings of fruits and vegetables. As reported in a previous FITS 2008 study (22), these same parents reported that almost a third of toddlers and preschoolers did not eat any discrete fruit or vegetable on any given day. The transition to toddlerhood is a key period for parents, childcare providers, and health care providers to encourage consumption of fruits and vegetables, especially since repeated exposures to vegetables in later infancy can increase their acceptance by very young children (23).

Consuming sweets, especially sugar-sweetened beverages, should be discouraged for infants and young children (7). The AAP recommends children not consume sugar-sweetened beverages because these beverages provide calories with minimal nutritional value and are associated with risk of childhood obesity and dental caries (7,9). Pediatricians, health care providers, and nutritionists play a vital role in improving parents’ understanding of what a healthy diet consists of for their child at different stages of development and by giving parents specific information on how to achieve a healthy diet for infants and young children (1,2,24,25).

Screen time is a risk factor for obesity because it entails no physical activity. Television viewing increases a child’s exposure to advertising for unhealthy foods (1,26). A child’s screen time can also displace play time, interaction with peers and family, and reading (27,28). The FITS 2008 showed that most toddlers and preschoolers were involved in an athletic activity or played outside 1 or more hours a day; however, nearly all toddlers and one-fourth of preschoolers engaged in more screen time than AAP recommended for their age. Longitudinal studies show that children who watch at least 3 hours of television daily at age 2 were almost 3 times as likely as other children to watch at least 3 hours per day at age 6 (27). A national prospective study found that 41% of 5.5-year-olds had a television in their bedroom and that this practice was associated with sleep problems (29). Data from the FITS 2008 show that 1 in 4 preschoolers had a television in their bedroom by age 4.

The FITS 2008 design and study limitations are described elsewhere (12). In brief, response rates were lower in FITS 2008 than in FITS 2002, consistent with a general downward trend in the US population’s participation in telephone surveys (15). As a result, our study sample may not be representative of children living in households with low levels of parental education. The weights used for analysis adjusted for the mother’s race/ethnicity and age to reflect the US population from birth to 4 years. Finally, although the data are proxy-reported, they are based on validated methods for estimating the food and nutrient intake of groups and reliable survey methods. These considerations should be taken into account when interpreting the findings.

FITS 2008 data indicate that toddlers and preschoolers are already developing some of the unhealthy habits seen in older children and adults and are not meeting important recommendations aimed at preventing obesity. The data also indicate that parents may not be aware of factors that put their child at risk of obesity later in life, such as failure to recognize when their child is overweight. The early years are when parents and caregivers must instill healthful, long-term dietary and physical activity behaviors in their children and family.

In 2012, the Institute of Medicine recommended evidence-based ways that parents, health care providers, and childcare providers can improve young children’s food and beverage environments and physical activity environments (2). Pediatricians and other health care providers are an important part of community plans to prevent obesity (2,24). They can help parents instill in their children healthful, long-term dietary and activity behaviors by 1) providing specific and targeted information on which behaviors are desirable and 2) explaining how to encourage their children to adopt such behaviors. But health care providers cannot do it alone. Changing diet and physical activity requires recognizing risk factors and a mutual effort by health care providers, parents, families, and communities to increase awareness and to support teaching healthy behaviors that will prevent obesity. FITS 2008 data inform the target areas for obesity prevention so that strategies can be identified through research and evaluation and then implemented to improve the environments where children live and play.
